# Genome sequences of *Staphylococcus aureus* RN4220

**DOI:** 10.1128/mra.00462-24

**Published:** 2024-12-16

**Authors:** Eon-Min Ko, Seonghan Kim

**Affiliations:** 1 Division of Bacterial Disease Research, Center for Infectious Disease Research, National Institute of Infectious Diseases, National Institute of Health, Korea Disease Control and Prevention Agency, Osong, South Korea; University of Maryland School of Medicine, Baltimore, Maryland, USA

**Keywords:** Whole-genome sequencing, *Staphylococcus aureus*

## Abstract

In this study, we present whole genome sequences of *Staphylococcus aureus* RN4220. Using PacBio DNA sequencing and *de novo* assembly, we obtained a single contig of 2,697,647 bp, which includes 2,652 predicted genes comprising protein-coding sequences, tRNAs, and rRNAs.

## ANNOUNCEMENT

*Staphylococcus aureus* is a prominent human pathogen, known for its versatile virulence mechanisms and ability to develop antibiotic resistance, which significantly challenges healthcare systems globally (1, 2). *S. aureus* RN4220 is a valuable model strain extensively used in research due to its well-characterized genetics and tractability for genetic manipulation (3). While previous studies have sequenced the genome of *S. aureus* RN4220 using hybrid approaches, a complete and detailed genome sequence using PacBio technology has not yet been reported (4, 5). Here, we provide a comprehensive genome sequence of *S. aureus* RN4220 using PacBio sequencing, emphasizing the methodology and the resulting genomic insights.

The *S. aureus* RN4220 strain was purchased from BEI Resources in 2009. Since acquisition, the strain has been stored at −80°C, supplemented with 40% glycerol. *S. aureus* RN4220 was cultivated in tryptic soy broth at 37°C with shaking at 200 rpm until reaching the late logarithmic phase of growth. Genomic DNA was isolated using the *Quick*-DNA Fungal/Bacterial Miniprep Kit (Zymo Research, Irvine, CA) following the manufactures’ instructions. For PacBio library preparation, the genomic DNA was not intentionally sheared; the DNA was size-selected using the BluePippin system to enrich for fragments larger than 20 kb. The SMRTbell Express Template Prep Kit 2.0 (Pacific Biosciences, Menlo Park, CA) was used to generate a library. The PacBio DNA Sequencing Kit 4.0 and 8 SMRT cells were used for sequencing. Sequencing was performed on the PacBio Sequel II platform, capturing 240-minute movies for each SMRT cell. A total of 5,358,237 reads were generated by sequencing, with an *N_50_* value of 9,491. Read quality control, error correction, and adapter trimming were performed using the SMRT Link software (version 8.0). The reads were assembled *de novo* using CANU (v2.2) (6). Genome assembly of the reads resulted in one contig comprising 2,697,647 bp with a GC content of 32.8%. The genome coverage achieved was 6,918. For circular genomes, overlaps were identified and trimmed to form a contiguous sequence. The genome was rotated to position the origin of replication at the start. Protein coding (CDS), tRNA, rRNA genes, and repeat regions were predicted by NCBI PGAP (Prokaryotic Genome Annotation Process), which uses RefSeq and HMM Library (TIGRFAM, Pfam, and PRK HMMs) databases (7, 8). Gene prediction and annotation of the genome resulted in 2,652 gene predictions (2,570 CDSs, 59 tRNAs, and 19 rRNAs) and 2,565 gene annotations (872 symbol, 485 EC_Number, 2,559 RefSeq, and 6 HMMs). The alignment of our data with the most recently published genome sequence (CP101124) revealed 35 mismatches and 5 gaps.

## Data Availability

The GenBank accession number for the *S. aureus* RN4220 genome sequences is CP150127. Biosample accession number is SAMN 40597727. The raw reads have been deposited in the NCBI Sequence Read Archive under accession number SRR28456670.
